# Book Notices

**Published:** 1882-02-20

**Authors:** 


### *BOOK NOTICES.*

CACTUS, OR THORNS AND BLOSSOMS.—We have read a copy of this beautiful volume of poems, by Mrs. Elizabeth O. Dannelly. The gifted authoress is a native Georgian, though for many years a resident of Baltimore, and from girlhood has been a valued contributor to various magazines and other periodicals of choice literature. This volume consists of religious, temperance, memorial and miscellaneous poems written in a singularly chaste, sprightly and graceful style, and some of the poems are remarkably beautiful, while others are vivid with the fire of eloquence. The poems are not only beautiful but moral in tone and sentiment, tending to cultivate the heart, and to elevate the thoughts and feelings of the reader.

The authoress is the widow of a Confederate Surgeon, and this, aside from her personal and literary worth, should commend her to the kindly notice of our professional brethren, and we hope the members of the profession will recommend the work to their friends and acquaintances, especially to the ladies. Publishing & Engraving Co., New York. Price, $1.50. P.

PLAIN FACTS FOR OLD AND YOUNG. By J. H. Kellogg, M. D., Member of the American Public Health Association; American Society for the Advancement of Science; American Society of Microscopy; member of Michigan State Board of Health; Medical Superintendent of the Battle-Creek Sanitarium; Author of numerous works on Health, etc. Published by Segner & Condit, Burlington, Iowa.

The above work treats of the interesting subjects of the "Sexual Relations; Marital Excesses; Prevention of Conception; Infanticide and Abortion; the Social Evil; Solitary Vice; Chapter for Boys; a Chapter for Girls", etc. It is a large work, containing over 500 pages, and though adapted to the popular reader, will be read with interest by the physician. It is moral in tone, and written in a style calculated to do good, very superior to the trashy books which have been published upon these subjects by a class of Quacks who play upon public credulity for the sake of gain only.

ARTIFICIAL ANÆSTHESIA AND ANÆSTHETICS. By Henry M. Lyman, A. M., M. D., Professor of Physiology and diseases of the Nervous System in Rush Medical College, Chicago; and Professor of Theory and Practice of Medicine in the Woman's Medical College, Chicago, Ill. New York: Wm. Wood & Co. Octavo, 338 pp.

The work enters fully into the subject of anæsthesia in all its phases, giving sphygmographic illustrations of the several anæsthetic agents as shown in experiments upon the lower animals. It is a work of much interest and of great practical importance.

A TREATISE ON DIPHTHERIA. By A. Jacobi, M. D., Clinical Professor of Diseases of Children, in the College of Physicians and Surgeons, New York; Physician to Bellevue, Mount Sinai and the German Hospitals, etc. New York: Wm. Wood & Co., 27 Great Jones St., 1880.

An octavo of 250 pages. A very thorough work on the subject of diphtheria, and should be read by every physician.

A TREATISE ON FOOD AND DIETETICS, PHYSIOLOGICALLY AND THERAPEUTICALLY CONSIDERED. By F. W. Pavy, M. D., F. R. S., Fellow of the Royal College of Physicians; Physician to and Lecturer on Physiology at Guy's Hospital. Second edition. New York: Wm. Wood & Co. 1881. P. 402, oc. McGarity & Laird, Agents, Atlanta.

The rapid exhaustion of the first edition and the call for a second, indicates the value of this work. We regard it a very able and valuable book, and one which will be appreciated by the intelligent and progressive practitioner.

